# Weathering in a world without terrestrial life recorded in the Mesoproterozoic Velkerri Formation

**DOI:** 10.1038/s41467-019-11421-4

**Published:** 2019-08-01

**Authors:** Mehrnoush Rafiei, Martin Kennedy

**Affiliations:** Department of Earth and Planetary Sciences, Macquarie University, Sydney, 2109 Australia

**Keywords:** Biogeochemistry, Ocean sciences

## Abstract

Today the terrestrial surface drives biogeochemical cycles on Earth through chemical weathering reactions mediated by the biological influence of soils. Prior to the expansion of life on to land, abiotic weathering may have resulted in different boundary conditions affecting the composition of the biosphere. Here we show a striking difference in weathering produced minerals preserved in the Mesoproterozoic Velkerri Formation. While the bulk chemistry and mineralogy is dominated by illite similar to many modern mudstones, application of a novel microbeam technology reveals that the initial detrital minerals were composed of mica (28%) and feldspar (45%) with only a trace amount (<2%) of typical soil formed clay minerals. The majority of illite and the high Al_2_O_3_ fraction previously interpreted as a weathering signal, is present as a replacement of feldspar and mica. These sediments record physical erosion with limited pedogenic clay mineral formation implying fundamentally different weathering pathways.

## Abstract (web summary)

Prior to the expansion of life on to land, abiotic weathering may have resulted in different boundary conditions affecting the composition of the biosphere. Here the authors studied clay minerals from a Precambrian rock record to reveal the weathering processes and find difference in weathering produced minerals preserved in the Mesoproterozoic Velkerri Formation.

## Introduction

Clay minerals in shales and mudstones provide a physical record of weathering reactions regulating the composition of the biosphere^1-6^. They are a major sink of weathering produced ions^7,8^, and are implicated in the burial efficiency of organic carbon in continental margin settings^9–11^. The flux of detrital clay minerals (DCM) varies through geologic time recording different tectonic or climatic conditions^12,13^. A fundamental stepwise increase is hypothesized to coincide with an increase in weathering effects associated with the expansion of simple life forms (e.g., microbes, lichens, fungi, liverworts) on to land forming the first soils^7,14–21^. Today, biologically active soils are the major locus of clay mineral production^1,13,22^. They accelerate regolith weathering and create a clay mineral factory by raising the ionic composition of pore fluids, retaining water, and producing organic acids and chelating compounds^15,23–25^.

The late colonization of Earth’s terrestrial surface by simple life forms^16,26^, followed by plants which currently make up ~80% of the Earth’s biomass^27^, implies that soil processes have not always exerted the same influence on the biosphere^28,29^ and that prior to this greening geochemical feedbacks associated with weathering were likely different^12,30–34^. Soils produce the vast majority of DCM, and DCM are the most common sedimentary mineral type in general comprising >60% of shales and mudstones^8,22,35^. The initiation of the soil ‘clay mineral factory’ should thus be a conspicuous addition to fine-grained sediments. However, the evident abundance of shale and mudstone units in Precambrian successions with similar composition to Phanerozoic mudstone argues against this expectation^36,37^. In the absence of soil production, an equivalent source of clay minerals recorded by these ancient mudstones remains an open question.

Here we directly determine the composition and origin of individual clay minerals in the Mesoproterozoic Velkerri Formation (see Methods). The Velkerri Formation was selected for this study because it is a well preserved, (pre-soil) clay-mineral rich, black shale that is commonly used as an informal reference section for Mesoproterozoic environmental conditions^30,38–42^. Although there is no single Formation capable of recording a comprehensive global view of weathering processes, the Velkerri Formation captures a broad regional pattern of weathering and is a reasonable basis for exploration of weathering processes in this time period.

We applied a novel method of analysis called Nanomin to identify the clay minerals present and their origin. Nanomin seamlessly integrates nanometer scale high-resolution microbeam imaging with quantitative mineral mapping determined by micro-X-ray scanning combined with a mineral deconvolution algorithm. It is both capable of imaging and identifying single clay crystallites and stitching together high-resolution quantified mineral maps up to 2 cm^2^ that bridge nanoscale to macroscale features. This approach complements previous studies of the integrated weathering signal from bulk sediment properties such as the chemical index of alteration^36,43^, secular changes of stable minerals^18^ phyllosilicates^8,35^, or geochemical proxies^12,30,34,40,41,44^ by focusing on the elemental composition and crystallinity of individual clay minerals that reveal the provenance, biologic, hydrologic, temperature and diagenetic conditions they formed within^13,22,45^.

We show that Nanomin quantitative mineral mapping of the Velkerri Formation offers a highly detailed view of an ancient landscape dominated by physical weathering conditions strikingly different from modern shales. It identifies clay minerals to have formed during extensive replacement of primary silicate minerals and within pores. Backtracking to the primary detrital composition of grains identifies a dominance of feldspar, mica, and quartz with only trace amounts of detrital clay minerals, reflecting the limited influence of soil weathering processes in the provenance region.

## Results

### Lithology

**Fig. 1:**
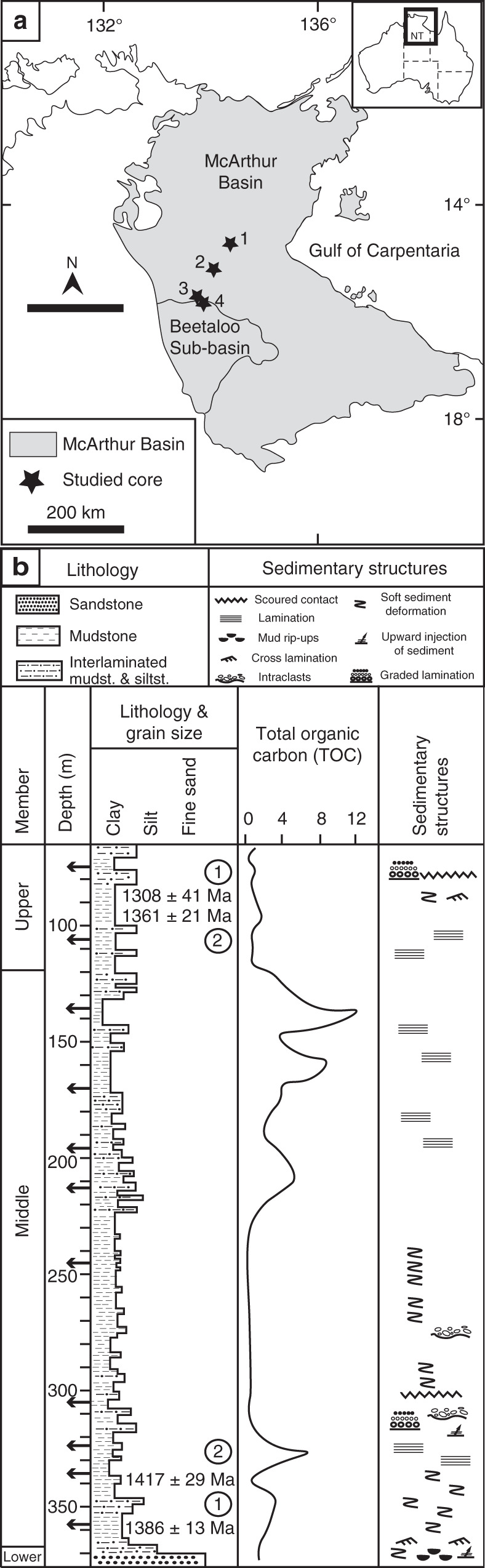
Location of the study area and sedimentary log of the Urapunga-1 core. **a** Location of the McArthur basin and studied cores (1: Urapunga-4, 2: Lady Penhryn-2, 3: McManus-1, 4: Walton-2). **b** log of the Urupunga-4 core for the interval housing the Velkerri Formation used as a type section in the absence of surface outcrop. Arrows indicate samples used (modified from^61^; depositional age of the Velkerri Formation after Yang et al.^57^ for 1 and Kendall et al.^40^ for 2)

**Fig. 2:**
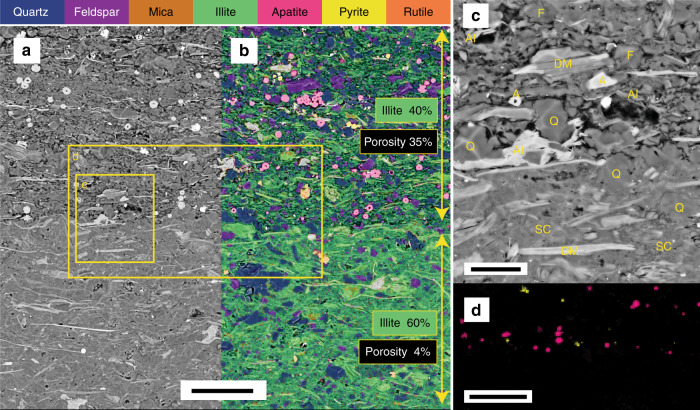
SEM backscatter image with Nanomin overlay of two laminae from McManus-1. **a** SEM backscatter image revealing textural difference and highlighting porosity (35% of image area) in the upper laminae vs. the occluded porosity (4% of image area) in the lower image. Grain size of detrital material is similar, but diagenetic pyrite and apatite are confined to the uncemented laminae. Scale bar = 40 μm. **b** Nanomin mineral map overlain on the continuation of the image in a to reveal the mineralogy showing 40% illite in the upper laminae vs. 60% illite present as interstitial quartz and illite cement in the lower laminae. **c** Detail of the contact between the laminae within box c reveals that the primary intergranular porosity in the upper laminae is largely preserved except in some areas where it is filled by minor authigenic clay crystals, apatite (A) and pyrite. Porosity in the lower lamiae is largely occluded by illite and silica (chert) cement (SC). Secondary mouldic porosity resulting from the dissolution of feldspar (F) in the cemented laminae postdates formation of cement with subsequent growth of authigenic illite (AI) within it. (DM detrital mica, Q quartz). Scale bar = 10 μm. **d** Mineral map of only apatite and euhedral pyrite with no underlying BSE layer identifies these minerals are limited to the open porosity laminae and implies a later timing of formation following the reduction of porosity in the lower laminae. Scale bar = 40 μm

**Fig. 3:**
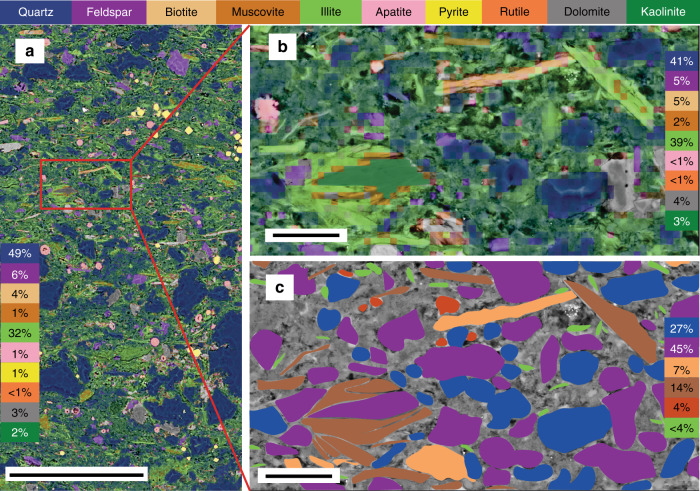
SEM backscatter image with Nanomin mineral map overlay from a dark fine-grained lamina in Urapunga 4 core. **a** A mineral map showing the distribution and composition of grains with quantified percentage of weight percent calculated from area showing quartz and illite to be the dominant mineral phases. Scale bar = 100 μm. **b** Zoomed in image from the box in a provides detail of the detrital silt grains, replacement minerals and intergranular micro quartz cement with minerals quantified for this area as in a. Scale bar = 10 μm. **c** Primary detrital constituent map of image b with replaced areas returned to likely original phases and intergranular cement left as background BSE image. Correction is based on relict crystal forms evident in BSE. Quantified normalized values for the area show feldspar is dominant with quartz, mica, and only a small component of detrital illite. Illite present in replaced mica and feldspar is 32% of the 39% in image b leaving a maximum of 7% that could have been deposited as DCM. C is a micaceous arkosic siltstone with a calculated CIA value of 58% indicative of low chemical weathering. The original sample from the core was taken from the finest-grained laminae, has a significant illite fraction (32%) and a CIA index of 73% suggestive of intense weathering resulting from illite replacement of feldspar and mica after deposition. Scale bar = 10 μm

**Fig. 4:**
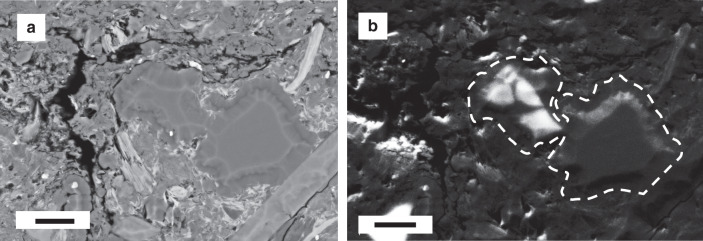
SEM backscatter and cathodoluminescence images of silica overgrowth cement around quartz grains. **a** SEM backscatter image showing two 25 µm quartz grains surrounded by clay sized minerals from Urupunga-4. **b** SEM cathodoluminescence image illustrating 3 to 4 angular quartz grains that have strong luminescence indicating a detrital origin^46^ with non-luminescent silica cement forming overgrowths that appear as silt sized grains. Scale bar = 10 μm

At macro scale, the Velkerri Formation is a conspicuously laminated black shale that varies in cementation. In the upper and lower portions of the core, laminae are defined by rippled silt and darker colored clay drapes (Fig. 1). Greater frequency of dark laminae equates with finer grained intervals and likely record a greater component of deposition from suspension vs. tractional current. In the middle of the formation laminae are most commonly parallel and more cryptic in composition because of the finer grain size. BSE imaging shows 40–100 µm laminae can be defined by both depositional and diagenetic phases including euhedral (digenetic) and framboidal (depositional) pyrite, kerogen (depositional) and bitumen (migrated), quartz silt (depositional) and quartz cement (diagenetic), mica flakes (depositional) and pore occluding illite, smectite and kaolinite (diagenetic) (Fig. 2). Laminae are commonly defined by abundance of cement rather than grain size or mineralogy (Fig. 2c). In general, the Velkerri Formation is well cemented with much of the core presenting as hard cherty rounds interspersed with less cemented friable intervals that have collapsed into chips and fragments. Cement is comprised of µm -scale quartz crystals (chert) (Fig. 3b, c) that can also form between authigenic illite crystals presenting a mixed quartz and illite cement (Fig. 2c). Cementation greatly reduces intergranular porosity, with a drop from 35 to 4% (based on image area) in adjacent laminae defined by the presence vs. absence of pervasive cement (Fig. 2c). Quartz cement also occurs by over-growth of detrital quartz grains to form silt-sized grains visible using the luminescence differences between detrital and authigenic quartz (Fig. 4)^46^. The bulk mineralogy of the Velkerri Formation includes quartz (53–45%), illite (55–45%), biotite and muscovite (2 M illite) 5–20%, albite, and K-feldspar (5–15%), kaolinite (<2%), pyrite (<5%). Glauconitic sands are common in some intervals as is diagenetic dolomite and mineralized pyrite and chalcopyrite^39,41,44,47^ although these intervals were not sampled in this study.

### Detrital vs. diagenetic minerals

Electron imaging in BSE combined with mineral mapping using Nanomin provide the spatial associations and composition revealing the detrital vs. diagenetic origins of these minerals. The primary detrital fraction is recognized as grains larger than 5 µm, that are abraded, rounded or show broken margins, or are subparallel to laminae and coherent with or form the depositional fabric. By contrast, diagenetic phases are delicate fibrous crystals that grow in a radiating pattern or fill intergranular pores, fractures, dissolved and ghosted or partially dissolved regions of feldspar or mica, or are euhedral shapes confined to pores or irregular fractures that cross cut primary laminae (Fig. 3). Grain size is typically <2 µm in the case of clay minerals. Diagenetic quartz forms as µm scale crystals that can coalesce to connect pores with stringers of cement up to 500 µm in length, or overgrow detrital grains to produce silt-sized grains of 20–40 μm (Fig. 4). It can occur both in association with intergrown illite (Fig. 2b) and without it (Fig. 2c).

**Fig. 5:**
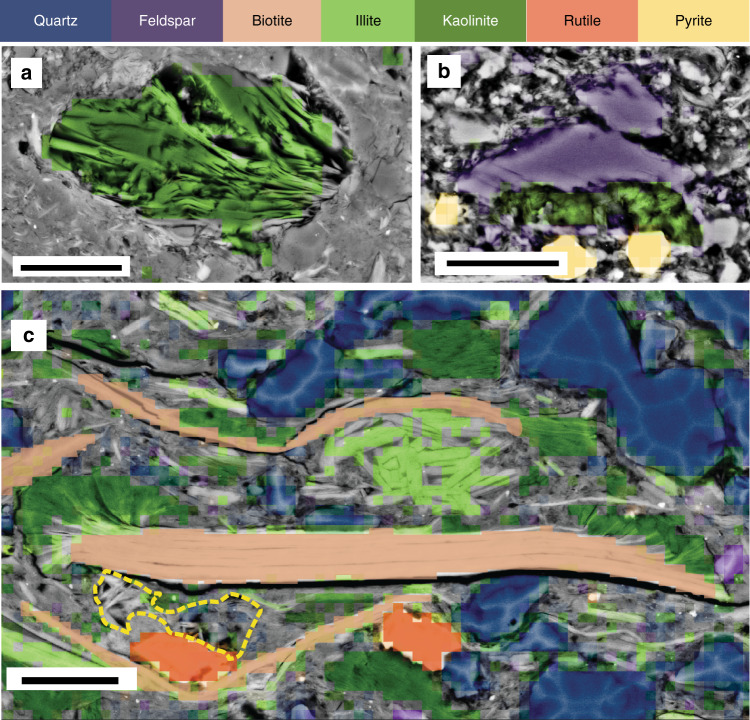
SEM backscatter images overlain by a Nanomin mineral map illustrating mineral replacement by kaolinite and illite. **a** Only kaolinite selected in the mineral map layer overlay showing euhedral kaolinite crystals formed within a 40 µm void most likely formed by feldspar dissolution (from the Urapunga-4 core). **b** albite, pyrite, and kaolinite selected in the mineral map overlay showing partial dissolution of a 20 µm feldspar crystal with authigenic kaolinite forming within the mouldic porosity. The boundaries of the minerals identified by Nanomin mineral mapping and the characteristic shapes of the minerals evident in BSE provide qualitative sense of the precision and accuracy mineral assignments (from the Walton-2 core). **c** A 70 µm biotite flake with alteration of the ends to kaolinite and illite. Illite replacement of smaller detrital mica minerals including a fan of small 2–4 µm crystals forming a floccule squeezed between the two large mica flakes. Detrital quartz grains sutured together by quartz overgrowth, and detrital rutile also evident. Dashed yellow line outlines an area of secondary porosity formed by feldspar dissolution with hairy, authigenic illite/smectite forming within it (from the Lady Penhryn-2 core). Scale bar = 10 μm

The mineralogy of the detrital fraction is comprised of quartz, mica, feldspar, illite, and trace amounts of rutile and pyrite <5 µm (Fig. 3). Detrital grains are unsorted to poorly sorted varying from thin mica flakes >120 µm long (Fig. 5c) to 5–10 µm illite crystals forming 15 µm floccules with diffusive boundaries (Fig. 5c). In non-cemented intervals, grain size is primarily silt with primary intergranular porosity preserved (Fig. 2c). Illite as DCM (1 M illite) (<5 µm) are challenging to separate from illite as μm scale ground mica (2 M) petrographically, but when DCM is present, it occurs between silt sized grains as small tabular crystals that are stacked parallel to bedding or deformed around quartz or feldspar grains (Fig. 2c). Quartz and feldspar grains are commonly <20 µm, and are angular to sub-rounded. Framboidal pyrite <5 μm is present in some samples where it is randomly dispersed and likely to be of pelagic origin.

The diagenetic fraction is dominated by micro-quartz crystals intimately associated with <3 µm scale illite crystals that form a pervasive cement between silt-sized detrital grains. The grain size, delicate crystal terminations of the illite and quartz evident in BSE indicate this idiotopic texture is post-depositional. The irregular distribution of cemented intervals in the core and between laminae supports a geochemically open system to silica and alkaline rich fluids from late stage basin-derived connate or early seawater sources. Later stage, pore lining hairy <1 µm illite or kaolinite books grow within mouldic porosity formed by pervasive dissolution of feldspar (Fig. 5a, b) or between grains in less cemented laminae (Fig. 2c). Illite and kaolinite partially or completely replace biotite or muscovite flakes (Fig. 5c). Euhedral pyrite is common, and typically concentrated with organic matter and apatite spheres.

### Identification of initial composition

We estimated the original detrital mineral composition by restoring the areas of replaced minerals (feldspar and mica) and removing the post replacement minerals (diagenetic illite, kaolinite as well as the quartz cement in pores). The diagenetic contribution was calculated as the difference between the estimated detrital and the present composition determined by Nanomin maps. Figure 3 shows an example of recalculation of the feldspar, mica and quartz composition following subtraction of the diagenetic phases and reassignment of the original detrital mineral to the ghosted crystal boundary evident in the BSE image. In this example, illite composition in the area of interest (Fig. 3a) drops from 39 to <4% (Fig. 3c), quartz drops from 41 to 27% and mica minerals rise from 7 to 21% and feldspar increases from 5 to 45%. ~79% of the illite fraction is accounted for as replacement of feldspar or mica, leaving a maximum of 21% of the illite (<7% of the total sample) that may have originally been deposited as DCM. This analysis suggests that while illite and quartz now dominate the bulk composition and rock properties of this sample, the detrital composition of the sediment was a micaceous feldspathic siltstone with minor detrital illite.

### Chemical index of alteration

**Fig. 6:**
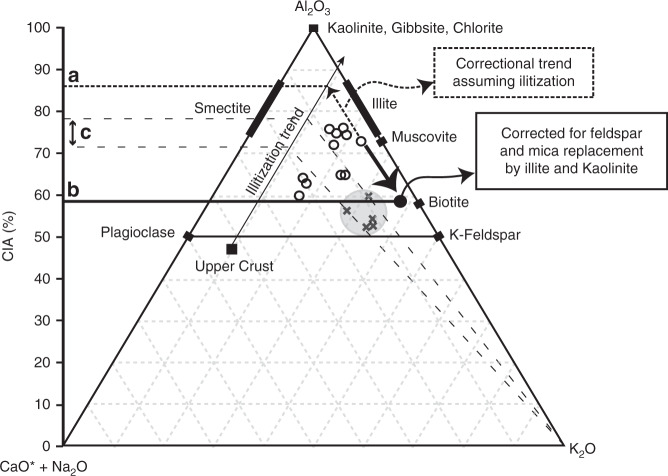
CIA ternary diagram plotting 11 samples (unfilled circles) range between 61 and 78% from laminated mudstones in the Urapunga 4 core. Elemental data from XRF analysis. Correction of CIA for K-metasomatism (illitization)^48^ following the dashed line (**a**) from the average upper crust value (black square) identifies a range of CIA values for these same samples up to 87%. Data from Tosca et al.^36^ (crosses) show a lower range of values between 51 and 61% that were corrected to 72–78% (**c**). The sample with highest corrected CIA value (from 107 m depth) was used to evaluate the effects of replacement and authigenic clay formation (Fig. 3). The CIA value for the restored detrital composition from Fig. 3c (filled circle) shows a lower CIA value (**b**) resulting from the addition of kaolinite and illite as replacement of biotite and feldspar. Average upper Archean upper crust composition identified by Condie et al.^62^ and adapted by Tosca et al.^36^ for correction for metasomatism

We determined CIA values for these samples to compare to the petrographic observations (see methods). Our results show a range of CIA values between 61 and 77 (Fig. 6; Table 1). We applied two different corrections to CIA values to account for possible diagenetic alteration (Fig. 6). Correction for illitization (metasomatism) using the method of Fedo et al.^48^ shows an increase in CIA to a maximum value of 87% (Fig. 6). We also used the petrographic-based estimate of the detrital fraction (detailed above and presented in Fig. 3) to calculate the CIA in the absence of the diagenetic phases to yield a range of values between 48–61%. These values are significantly lower reflecting the greater fraction of unweathered minerals and the removal from the calculation of the aluminous replacement phases. This lower value is consistent with the textural and mineralogical immaturity of the sediment.

**Table 1:** Calculation of CIA from XRF for 11 samples from the Urapunga 4 core’s laminated mudstones

Sample depth (m)	XRF analysis (mol)				
	Al_2_O_3_	Na_2_O	K_2_O	CaO*	CIA (%)
358.00	0.181	0.015	0.043	0.000	76.0
336.83	0.192	0.017	0.041	0.000	76.9
324.02	0.135	0.016	0.034	0.000	73.0
304.52	0.176	0.017	0.039	0.000	75.6
246.45	0.153	0.017	0.030	0.000	76.5
212.20	0.127	0.016	0.033	0.020	64.8
196.20	0.106	0.017	0.030	0.020	61.0
170.05	0.115	0.016	0.031	0.020	63.3
136.00	0.123	0.010	0.044	0.010	65.5
107.25	0.112	0.004	0.063	0.000	73.6
77.35	0.182	0.010	0.048	0.000	75.1

^a^Correction for carbonate and apatite made by using the method of ^63^ to estimate CaO for silicate fraction (CaO*)

## Discussion

Modern riverine sediment flux is dominated by clay minerals formed by soils. This may have been different in the past, particularly prior to the advent of life on land and the influence of the first soils. The close link between biology and weathering in soils evident today implies that evolutionary changes likely changed the nature of weathering reactions and thus the set point of biospheric conditions in the past^20,29,31–34^. Further, the influence of clay minerals on the burial of organic carbon may have had a direct effect and provided an important feedback on temperature and atmospheric oxygen levels^9,10^. A secular increase in clay minerals produced by the initiation of soil weathering has been suggested to drive a step increase in carbon burial and been a significant influence on the transition from the Precambrian to the Phanerozoic biosphere^19,29^. This hypothesis makes a simple prediction that the composition of Precambrian shales and mudstones should have a notable compositional difference from modern shale with a much-reduced component of pedogenic formed clay minerals and a greater component of physically weathered primary minerals. While ultimately chemical weathering of the crust must have balanced planetary emission of CO_2_^7^, the formation of clay minerals from weathering reactions is only one pathway in which CO_2_ is cycled^5,6,14,33,49–51^.

Siltstones and mudstones are seemingly abundant in ancient marine sediments that predate the first evidence of complex life on land and soils^8,35^ while being rare in terrestrial settings^28^. While the grain size fraction of mudstones is largely a function of transport that would not be substantially different in the past, the depositional mineral composition of these sediments is what may make them different from Phanerozoic mudstones. Determining this composition is notoriously difficult both because the µm grain size cannot be resolved with standard petrographic techniques so most studies use bulk sample approaches without benefit of contextual information, and clay minerals are highly reactive and undergo mineral and compositional change from their original depositional composition to a stable burial composition^4,45,46^. Past studies have characterized the gross mineralogical variability using powder X-ray diffraction that integrates depositional and post depositionally formed minerals to estimate weathering intensity^19,36^ from the detrital fraction. This study focuses on separating the depositional from the diagenetic component in order to better characterize the physical and chemical influences in the weathering environment (Fig. 3).

The abundance of illite and kaolinite within the Velkerri Formation has been taken as evidence for intense chemical weathering similar to that recorded in Phanerozoic mudstones considering these minerals as entirely detrital and unaltered^12^ or illitized replacement of an initially more smectitic detrital composition^36^. Kaolinite is metastable and Precambrian- aged detrital kaolinite is rare^52^ whereas formation of euhedral kaolinite from acid reactions in ancient organic rich rocks like the Velkerri Formation is not. While Cox et al.^12^ report 25% kaolinite in the Velkerri Formation interpreted as DCM, the minor amount of kaolinite we observed (<3%) is euhedral in form and diagenetic in petrographic context (Fig. 5), primarily replacing feldspar. Tosca et al.^36^. record no kaolinite in their samples.

Smectitic minerals are also metastable and typically undergo illitization^45,53^. Tosca et al.^36^. determined the polymorphs of illite in the Velkerri Formation and other Precambrian mudrocks to estimate the original detrital clay composition. Within the Velkerri Formation, they identified 9–15% 2 M (high temperature mica) with 18–30% 1 M and 1 Md (low temperature illite) polymorphs they interpreted as a replacement of detrital smectite or mixed layer Illite/smectite (I/S) during illitization. 1M-1Md Illite can, however, result from a number of different origins in addition to illitization of detrital I/S including replacement of feldspar or biotite, growth as a pore filling authigenic phase, or illite formed in soils and deposited directly as a detrital mineral^45^. The detrital minerals in the Velkerri Formation identified here are dominated by quartz, feldspar, and mica flakes (2 M) consistent with the composition from XRD reported by Tosca et al.^36^. The difference lies in the 1M-1Md illite fraction which is evident as an authigenic phase replacing detrital mica and feldspar (Fig. 5) and as a pore filling cement (Fig. 3) that comprises 78% of the non-mica (2 M) illite. While some smectite is present in the Velkerri Formation, petrographic relationship shows that it euhedral and formed late within the mouldic porosity associated with feldspar dissolution and does not form part of the depositional texture. After accounting for illite replacing primary minerals, detrital I/S could have made up a maximum of 7% of the total detrital composition. These samples differ substantially in texture from Phanerozoic mudrocks in which 40% illite of a detrital origin would form the primary fabric of the sediment comprised of clay platelets aligned with or defining laminae^4^. The degree of clay and quartz cementation apparent in these samples is common in sandstone reservoirs with high feldspar content^54^ reflecting both local and basinal sources of K^+^ and Si^+2^ ions. Cementation is also consistent with derivation of Si^+2^, K^+^ and Al_2_O_3_ ions by acid reactions associated with migrating hydrocarbons or mineralizing basin fluids^38,40^ or even early formed cement (precompaction) sourced from alkaline and silica saturated seawater.

The petrographically identified detrital fraction we observed presents a very different impression of the ancient weathering environment than that of most Phanerozoic shales. The striking assemblage of thin and delicate mica flakes and feldspar forming up to 45% of the sediment suggests that this sediment fraction was not the product of chemical but rather physical weathering. Feldspar and mica minerals split easily along cleavage planes and undergo rapid hydrolysis reactions typically resulting in them being a low percentage of Phanerozoic shale^13^. This fraction would be more concentrated in marine sediments in the absence of a significantly reduced soil formed detrital clay fraction. In this case weathering produced ions are not retained by the sorptive and concentrating properties of soils that promote clay mineral precipitation resulting in loss of ions within weathering solutions. Additionally, the absence of the stabilizing influence of biology on the landscape surface^28,55^ resulted in more rapid transfer and less physical and chemical weathering of primary sediment grains such as mica and feldspar.

High CIA values measured in the Velkerri Formation have also been used as evidence for similar weathering intensity to that recorded in Phanerozoic mudstones^12,36,52^. CIA values recorded in the Velkerri include 49–60%^36^ and 49–80%^12^ compared to our range 61–77%. The lower range of values for^36^ likely reflects sampling in coarser grained intervals with a lower phyllosilicate fraction in general than our samples as the composition of their samples contained up to 20% glauconitic sands compared to no glauconite in our samples. While their initial values record a low weathering intensity^36^, applied a correction^48^ for illitization and reported final values of 70–80% they interpreted to record high weathering intensity (see methods).

Our CIA data, while consistent with other studies, is not consistent with the detrital composition of the sediment determined petrographically. When we recalculate the CIA value after removing the late stage (burial) aluminous phases that replace primary detrital minerals, the CIA values drop to 40–50% (Fig. 6), consistent with the limited chemical weathering implied by the feldspathic and micaceous composition. In this case, a petrographically based correction provides an opposite effect on the corrected CIA value to the common correction^48^ for metasomatism. When the correction for metasomatism^48^ is applied to our data, it increases the CIA value up to 87% (Fig. 6). The difference between these corrections lies in the assumption that the present illite is entirely of a detrital smectitic origin vs. the petrographic evidence which shows the detrital fraction was unlikely to have ever comprised a significant fraction of the sediment.

The late stage formation of clay minerals in the Velkerri Formation explains the seeming contradiction between the abundance of phyllosilicates in an interval of Earth history that lacked the soil clay mineral factory. The primary mineral composition of these rocks indicates an igneous or metamorphic provenance dominated by physical stripping. This implies that the influence of terrestrial weathering on geochemical cycles likely varied to that of the present. While chemical weathering reactions must have balanced planetary emissions of CO_2_, clay mineral uptake of weathering produced ions in the Velkerri Formation was not as conspicuous sink of alkalinity. Instead, weathering produced ions took different pathways^20,50^ defining different boundary conditions for the biosphere. The evidence presented here is consistent with the displacement of weathering produced ions from clay minerals formed in soils to clay minerals formed from precipitation of silica saturated and alkaline seawater as early diagenetic cements during reverse weathering reactions^5,6^. The reduction in the production and flux of clay minerals would also have reduced the influence clay minerals exert on carbon burial today^9,56^ affecting temperature and oxygen concentration.

## Methods

### Samples

The Velkerri Formation is a deep water, organic rich (10% total organic carbon, TOC) mudstone to siltstone forming part of the Roper Group. The Roper Group varies from 1000–5000 m in thickness. It was deposited as a laterally persistent sheet (>145,000km^2^) across much of northern Australia in the post rift phase of the MacArthur Basin^44,47,57,58^ from 1361 ± 21 Ma to 1417 ± 29 Ma based on Re/Os dating^40^ or <1308 ±41 ma from detrital zircon^57^. Based on zircon ages, trace metals, and abundance of plagioclase, sediment records an increase in mafic source influence from the Mt Isa to Arunta region >400 km to the south^57,12,30,57^. The Velkerri Formation itself has experienced limited deformation and is recognized as a series of parallel reflectors across the MacArthur basin in seismic profiles^59^. It is shallowly buried and unmetamorphosed, and of a low thermal maturity, entering or within the oil generation window including live oils shows^42,47^. This study utilized 26 samples from the Urupunga-4, McManus1, Lady Penrhyn 2 and Walton 2 cores spanning ~200 km across the MacArthur basin in Northern Australia (Fig. 1). The Velkerri Formation is ~330 m thick in the Urupunga-4 reference section (Fig. 1) with a uniformity of appearance between cores suggesting limited local tectonic influence with deposition in a broad, lower energy, contiguous basin below storm wave base. Interbedded siltstone and fine sandstone intervals do show evidence of weak bottom currents that includes subtle scoured bases, ripples, glauconitic sands, and grading 45. The organic carbon-rich facies (>10% TOC) in the middle member shows a greater fraction of finely laminated mudstone intervals while lacking evidence of reworking by tractional currents or winnowing indicating deposition from suspension of pelagic or hemi-pelagic sourced sediment 45. We targeted these finest-grained dark-colored, parallel <mm laminated intervals for sampling to capture the highest proportion of DCM. We avoided intervals with evidence of tractional currents or grading that might indicate winnowing of clay minerals and concentration of coarser grained sizes more representative of current sorting and less likely to capture the weathering signal from DCM.

### Mapping shale mineralogy

Argon ion milled and carbon coated ultra-polished blocks were imaged using a FEI Teneo Field Emission Scanning Electron Microscope which is equipped with secondary and backscattered-electron detectors and an integrated Bruker Energy Dispersive X-ray Spectroscopy (EDX; Bruker XFlash Series 6) analyzer. Mineral abundance is determined by the Nanomin system using EDX data collected at 500 nm to 1 µm pixel resolution. Nanomin is designed specifically for fine-grained sediments, such as shales and differs from other automated mineral mapping systems by enabling deconvolution and identification of potential minerals that form mixed phase X-ray spectra allowing up to three mineral phases to be modeled for each pixel. Mineral mapping was performed using the FEI Maps2.0 Mineralogy Software that collected BSE and EDS spectra within high resolution tiles and stitched them together to construct composite images of up to 3 cm in diameter. Minerals are only broadly classified in this study to avoid interpretations dependent on minerals with similar spectra or within a continuous series. For example biotite and muscovite are grouped as mica in some analyses. The mineral illite is the dominant phyllosilicate phase in these samples. It is often defined as the clay sized fraction of mica^13^ however here we separate it from mica based on the Al, Ca, Fe, Mg, and Na ratios. In many cases, distinctive mica mineral shapes have an illite composition reflecting diagenetic alteration. Our imaging and mineral mapping data show that smectite is minor (<2%) and limited to pore lining authigenic phases consistent with trace amounts measured with X-ray diffraction. We have not specified smectite in the data reported here.

The precision and accuracy of Nanomin mineral identification and quantification can be evaluated by comparing the mineral boundaries assigned by Nanomin to the mineral boundaries independently defined by density difference evident in the spatially co-located Back scatter electron image (BSE). For example, the difference in contrast identifying the diagnostic crystal forms of kaolinite, mica, pyrite or feldspar evident in BSE (Fig. 5) closely coincide with the boundaries defined by X-ray determined elemental composition used by Nanomin. The pixelated margins of the mineral in the mineral map identifies the limits of resolution, while the identification of the specific minerals can be compared to the characteristic BSE mineral form to assess the accuracy and completeness of mineral mapping. The percentage of minerals reported is based on the area of the image that can also be converted to weight percent using standard mineral properties. Specific areas of interest can be selected with Nanomin and quantified to explore the compositional differences of features such as laminae. Single elements or minerals can be isolated and mapped to identify their spatial pattern or mapped with a BSE background layer to identify unknown BSE minerals (Fig. 5). Minerals smaller than the 500 nm resolution were identified by comparison of larger regions with similar mineral shapes evident in BSE with EDS spot analysis standardly done in microbeam studies^60^.

### Measurement of chemical index of alteration

To measure the elemental composition used to calculate the chemical index of alteration (CIA), we used a PANalytical Axios 1 kW X-ray fluorescence instrument (XRF). The CIA formulated by Nesbitt and Young^43^ is a proxy for the degree of chemical weathering recorded by detrital clay minerals. The formula [(Al_2_O_3_/Al_2_O_3_ + Na_2_O + K_2_O + CaO*) × 100], where CaO* is only the amount of CaO associated with the silicate fraction of the rock and excludes carbonates and apatite. The CIA index does not discriminate between detrital and diagenetic contribution of elements; however, it is commonly corrected for K^+^ addition assumed to occur with illititization of detrital clays with an initially lower K/Al_2_O_3_ composition like smectite^48^. In this correction K^+^ is not assumed to be limiting and sourced from and external fluid in a geochemically open system. K^+^ is scaled along a line within a ternary diagram (Fig. 6) until it is proportional with Ca^+2^ and Na^+^ in average upper crust for the age of sediment being measured. This correction assumes that the K:Ca + Na ratio of clay minerals formed during weathering had an initial ratio of the average crust, that all clays in the sample are of a pedogenic origin, and that there is no contribution from authigenic clays or loss of elements by feldspar replacement.

## Data Availability

The authors declare that the data used in this study are available within the paper.
