# Author Correction: Weathering in a world without terrestrial life recorded in the Mesoproterozoic Velkerri Formation

**DOI:** 10.1038/s41467-020-16614-w

**Published:** 2020-06-05

**Authors:** Mehrnoush Rafiei, Martin Kennedy

**Affiliations:** Department of Earth and Planetary Sciences, Macquarie University, Sydney, 2109 Australia

**Keywords:** Biogeochemistry, Ocean sciences

Correction to: *Nature Communications* 10.1038/s41467-019-11421-4, published online 1 August 2019.

The current sentence in Acknowledgement reads as “Financial support for this work was made available by the Australian Research Council LP12020086 and LE160100155”, and this should be corrected to “Financial support for this work was made available by the Australian Research Council LP12020086 and LE160100155 and by an Australian Government Research Training Program (RTP) Scholarship”.

